# Differential Effects of Camel Milk on Insulin Receptor Signaling – Toward Understanding the Insulin-Like Properties of Camel Milk

**DOI:** 10.3389/fendo.2016.00004

**Published:** 2016-01-27

**Authors:** Abdulrasheed O. Abdulrahman, Mohammad A. Ismael, Khaled Al-Hosaini, Christelle Rame, Abdulrahman M. Al-Senaidy, Joëlle Dupont, Mohammed Akli Ayoub

**Affiliations:** ^1^Biochemistry Department, College of Science, King Saud University, Riyadh, Saudi Arabia; ^2^Department of Pharmacology and Toxicology, College of Pharmacy, King Saud University, Riyadh, Saudi Arabia; ^3^UMR7247, CNRS, Nouzilly, France; ^4^Université François-Rabelais, Tours, France; ^5^L’Institut Français du Cheval et de l’Équitation, Nouzilly, France; ^6^UMR85, Biologie et Bioinformatique des Systèmes de Signalisation Group, INRA, Unité Physiologie de la Reproduction et des Comportements, Nouzilly, France; ^7^LE STUDIUM^®^ Loire Valley Institute for Advanced Studies, Orléans, France

**Keywords:** camel milk, insulin, insulin receptor, BRET, GRB2, IRS1, ERK1/2, Akt

## Abstract

Previous studies on the Arabian camel (*Camelus dromedarius*) showed beneficial effects of its milk reported in diverse models of human diseases, including a substantial hypoglycemic activity. However, the cellular and molecular mechanisms involved in such effects remain completely unknown. In this study, we hypothesized that camel milk may act at the level of human insulin receptor (hIR) and its related intracellular signaling pathways. Therefore, we examined the effect of camel milk on the activation of hIR transiently expressed in human embryonic kidney 293 (HEK293) cells using bioluminescence resonance energy transfer (BRET) technology. BRET was used to assess, in live cells and real-time, the physical interaction between hIR and insulin receptor signaling proteins (IRS1) and the growth factor receptor-bound protein 2 (Grb2). Our data showed that camel milk did not promote any increase in the BRET signal between hIR and IRS1 or Grb2 in the absence of insulin stimulation. However, it significantly potentiated the maximal insulin-promoted BRET signal between hIR and Grb2 but not IRS1. Interestingly, camel milk appears to differentially impact the downstream signaling since it significantly activated ERK1/2 and potentiated the insulin-induced ERK1/2 but not Akt activation. These observations are to some extent consistent with the BRET data since ERK1/2 and Akt activation are known to reflect the engagement of Grb2 and IRS1 pathways, respectively. The preliminary fractionation of camel milk suggests the peptide/protein nature of the active component in camel milk. Together, our study demonstrates for the first time an allosteric effect of camel milk on insulin receptor conformation and activation with differential effects on its intracellular signaling. These findings should help to shed more light on the hypoglycemic activity of camel milk with potential therapeutic applications.

## Introduction

Since many years, the camel raises passions principally due to its fascinating capacity of adaptation in severe environmental and diet conditions as well as the composition and the therapeutic properties of its milk (1). Indeed, many studies reported interesting biochemical properties of camel milk (2–4) and its chemical composition (5–8), as well as the stability of its components compared to other mammals, such as human and bovine (2). Historically, camel milk was proposed as an alternative treatment for a number of medical problems (9) and to have potential benefits in many diseases, such as allergy and viral infections (10, 11). Nevertheless, the most important observations remains its hypoglycemic activity observed in type 1 diabetes using both human and animal models (11–18). For instance, camel milk was proposed as an adjunct to insulin-based therapy allowing the reduction of insulin doses required in patients with type 1 diabetes (12, 14, 19). This suggests that the anti-diabetic activity of camel milk is mediated by an insulin-like and/or immune-modulatory effects on beta-cells of the pancreas (17). The amino acid sequence of some proteins isolated from camel milk has been reported to be rich in half-cystine, which implies a similarity with insulin family of peptides (20). The direct effect of milk insulin itself is also possible since camel milk has been shown to contain high concentrations of insulin (2, 21) compared to what is found in cows’ milk (16) and this seems to depend on the lactation stage. However, if insulin in camel milk is involved, it is still unclear how it can stay biologically active after its absorption by the intestinal epithelium even though many possibilities have been evoked (22). Thus, many studies reported the beneficial effects of camel milk in diabetes mellitus but the cellular and molecular mechanisms involved in such effects are completely unknown.

In this study, we hypothesized that camel milk may have a “direct” effect on insulin receptor activation and function at the principal target tissues. To test this hypothesis, we examined the effect of camel milk on the activation of human insulin receptor (hIR) by investigating its physical association with two key signaling proteins, insulin receptor signaling proteins (IRS1) and growth factor receptor-bound protein 2 (Grb2), known to bind directly or indirectly to the receptor upon its activation/phosphorylation by insulin binding. For this, we used bioluminescence resonance energy transfer (BRET) technology as previously reported for insulin receptor (23, 24), epidermal growth factor receptor (EGFR) (25), and various G protein-coupled receptors (GPCRs) (26–28). This approach allowed us to assess the activation of hIR through the monitoring of IRS1 and Grb2 binding to the protein complex involving the receptor, in real-time and live cells, before and upon activation with insulin. Moreover, we attempted to link our BRET data with the hIR downstream signaling pathways by assessing insulin-induced phosphorylation of protein kinase B (or Akt) and extracellular signal-regulated kinases (or ERK1/2) known to translate the activation by hIR of IRS1 and Grb2, respectively (29, 30).

## Materials and Methods

### Mammalian Expression Plasmids

The mammalian expression plasmids coding for the different proteins used in this study were as follow: hIR fused with *Renilla* luciferase 8 (Rluc8) was a gift from Dr. Rasmus Jorgensen (Hagedorn Research Institute, Novo Nordisk, Gentofte, Denmark), the IRS1 (1–262)-YFP was kindly obtained from Dr. Tarik Issad (Cochin Institute, Paris, France), and Grb2–Venus was provided by Dr. Kevin Pfleger (Harry Perkins Institute of Medical Research and Centre for Medical Research, Nedlands, Australia). All the plasmids were sequenced and their correct expression was verified by luminescence and fluorescence in parallel to BRET measurements as described below.

### Cell Culture and Transient Transfection

HEK293 cells were cultured in Gibco^®^ Dulbecco’s modified Eagle’s medium (DMEM) supplemented with 10% fetal bovine serum (FBS), 100 units/ml penicillin, 0.1 mg/ml streptomycin, and maintained at 37°C in 5% CO_2_. For transient transfections, Lipofectamine^®^ 2000 reagent was used according to the manufacturer’s instruction (Invitrogen, Carlsbad, CA, USA). Per well of 96-well microplates, the total of 200 ng of plasmids (100 ng of hIR–Rluc8 + 100 ng Venus/YFP-tagged proteins) and 0.5 μl of Lipofectamine were separately pre-incubated in 25 μl serum-free DMEM media for 5 min at room temperature. Then both solutions were mixed and further incubated 20 min at room temperature before adding the mix to the cells. Cells were harvested with 0.05% Trypsin–EDTA and after neutralization with DMEM cells were counted using Countess^®^ Automated Cell Counter (Invitrogen, Carlsbad, CA, USA) and a density of 10^5^ cells per well of 96-well plates were seeded with the transfection mix and incubated 48 h before BRET measurements.

### Camel Milk and Its Fractionation

Fresh camel milk was obtained from local farms in Riyadh and different forms of milk have been used in this study: (i) simply defatted camel milk, (ii) whey versus casein fractions, (iii) milk protein fractions with different molecular weights, or (iv) protein versus non-protein fractions. Camel milk sample was defatted by centrifugation at 4000 rpm for 30 min at 4°C. For the separation of the whey components from caseins, acid precipitation at pH 4.6 was performed by addition of 10% (*v*/*v*) acetic acid followed by centrifugation at 4000 rpm for 30 min at 10°C. Moreover, in order to separate the different protein fractions of camel milk according to their molecular weight, the defatted camel milk was subjected to centrifugation at 3500 × *g* and 25°C for 60 min and then filtration using different Amicon Ultra-15 centrifugal filters; 10,000, 30,000, and 50,000 NMWL according to the manufacturer’s instructions (Millipore Corporation, Billerica, MA, USA).

### Gel Filtration Chromatography

For this, we used Sephadex G-25 column allowing a fractionation range for globular proteins of 1000–5000 molecular weight. Sephadex G-25 resin (15 g) was soaked in deionized water overnight at 4°C and then decanted to remove the fine particles that did not settle. The hydrated resin was transferred to PBS buffer, pH 7.4, for equilibration and packed onto a column (diameter, 2.6 cm; length, 40 cm; XK 26 chromatography column, GE Healthcare). The defatted camel milk (10 ml) was applied to the column and eluted with the PBS buffer. The flow rate was set at 1 ml/min and fractions of 3 ml were collected using a fast protein liquid chromatography system (AKTA purifier GE Healthcare, Uppsala, Sweden). The concentration of protein and that of peptides in the eluted fractions were monitored at 280 and 215 nm. The amount of proteins present in the milk samples and fractions was quantified by the method of Bradford [100 mg Coomassie Brilliant Blue G-250, 50 ml 95% ethanol, 100 ml 85% (w/v) phosphoric acid, and 850 ml distilled water] using bovine serum albumin (BSA) as standard.

### Sodium Dodecyl Sulfate-Polyacrylamide Gel Electrophoresis

Camel milk fractions were subjected to SDS-PAGE using 4% staking and 12% resolving polyacrylamide gels (18 mA/1 mm thickness gel), running 2 h in 0.5 M Tris–HCl pH 6.8 and 1.5 M Tris–HCl pH 8.8 buffers, respectively. Proteins were subsequently stained for overnight in a solution containing the mixture of 0.1% (*w*/*v*) Coomassie blue R-250, 40% (*v*/*v*) methanol, 50% (*v*/*v*) water, and 10% (*v*/*v*) acetic acid. The gels were de-stained in a solution containing the above latter mixtures but without the Coomassie Blue R-250.

### BRET Measurements

Forty-eight hours post-transfection, HEK293 cells initially cultured in 96-well white microplates to 10^5^ cells/well were first pre-treated or not with 100 μl/well of defatted camel milk or its fractions at 37°C. After wash with PBS 1×, adherent cells were resuspended into 60 μl/well of PBS and BRET measurements were carried out in a final volume of 100 μl/well upon addition of 20 μl of bovine insulin (Sigma-Aldrich, St. Louis, MO, USA) at the indicated concentrations and 20 μl of Coelenterazine-h substrate (5 μM final) (Promega, Madison, WI, USA) as previously shown (25–28). BRET recordings were performed in real-time and live cells using Mithras^2^ LB 943 Multimode Reader (Berthold Biotechnologies, Bad Wildbad, Germany) allowing the sequential integration of luminescence signals detected with two filter settings (Rluc filter, 480 ± 20 nm; YFP filter, 540 ± 25 nm). For the determination of the optimal time of camel milk pre-incubation, cells were first pre-incubated different times (0, 5, 10, 15, 30, and 60 min) with camel milk at 37°C and BRET signals were then measured upon addition of 100 nM of insulin. For the dose–response analysis, cells were first pre-incubated 30 min at 37°C with camel milk before BRET signals were recorded in the absence or presence of increasing doses of insulin (0.001, 0.1, 1, 10, 100, 1000, 10,000 nM).

### Luminescence and Fluorescence

The amount of Rluc8 and YFP/Venus fusion protein expressed was determined for each transfection condition. The relative luciferase activity of hIR–Rluc8 was determined in parallel to BRET measurements where 80 μl of cells in PBS were incubated with 20 μl of Coelenterazine-h substrate (5 μM final) and Rluc8 emission was then measured at 480 nm. For the fluorescence of YFP/Venus-tagged proteins, 100 μl of cells in PBS were plated in 96-well black microplate and fluorescence emission was then recorded at 535 nm after excitation of the cells at 480 nm using the Mithras LB 943 plate reader.

### ERK1/2 and Akt Phosphorylation

HEK293 cells transiently co-expressing hIR–Rluc8 and Grb2–Venus were used for ERK1/2 and Akt activation using the classical SDS-PAGE and western blotting technique as well as the homogenous time-resolved fluorescence (HTRF^®^)-based assay (CisBio Bioassays, Codolet, France) as previously described (31, 32), respectively. For SDS-PAGE and western blot, cells were first cultured in 6-well plate and starved overnight in serum-free DMEM. After pre-treatment or not with 1 ml/well of defatted camel milk for 30 min at 37°C, cells were then stimulated or not with 100 nM or 1 μM of insulin in 500 μl/well of PBS for 5 min at 37°C. Protein extraction and western blotting were then performed using the rabbit polyclonal antibody against phospho-ERK1/2 (Thr202/Tyr204) (Santa Cruz Biotechnology, Santa Cruz, CA, USA) diluted 1/1000. The western blot signals were quantified with GeneTools software (release 4.01.02) and expressed in arbitrary units after normalization. In HTRF^®^-based assay, the two-plate protocol was used as recently described (32) after pre-treatment or not with defatted camel milk and stimulation or not with 100 nM insulin at the indicated times. The plate were then incubated for 2 h at room temperature before reading the fluorescence emission at 620 and 665 nm using the appropriate HTRF programs on Mithras LB 943 plate reader.

### Data and Statistical Analysis

Data are presented in BRET ratio as previously described (33). For the experiments in the presence of camel milk, the effects are indicated as “Insulin-induced BRET” in “% of control” where the insulin-promoted BRET signals in cells non-treated with camel milk were taken as 100% of the response. The kinetic curves and the sigmoidal dose–response curves were fitted using Prism 5 graphing software (GraphPad, La Jolla, CA, USA) allowing the analysis according to non-linear regression with the following equations: BRET = BRET_0_ + (BRET_max_ − BRET_0_)/{1 + 10^[LogEC_50_ − (insulin)]} for dose–response curves [BRET signals in function of Log (insulin)], and BRET = BRET_max_*[1 − exp(−K*time)] for kinetics [BRET signals in ­function of time (minutes)]. The ERK1/2 and Akt data were represented as HTRF Ratio corresponding to the following ratio: (the emission at 665 nm/emission at 620 nm) × 10,000. One-way and two-way ANOVA analysis using Turkey’s multiple comparisons test was used to determine statistically significant differences between untreated (in the absence of camel milk/fractions) and treated (in the presence of camel milk/fractions) conditions. ***p < *0.001*, **p < *0.01*, *p < *0.05; ns; non-significant*.

## Results

### BRET to Monitor hIR Activation in Real-Time and Live Cells

**Figure 1:**
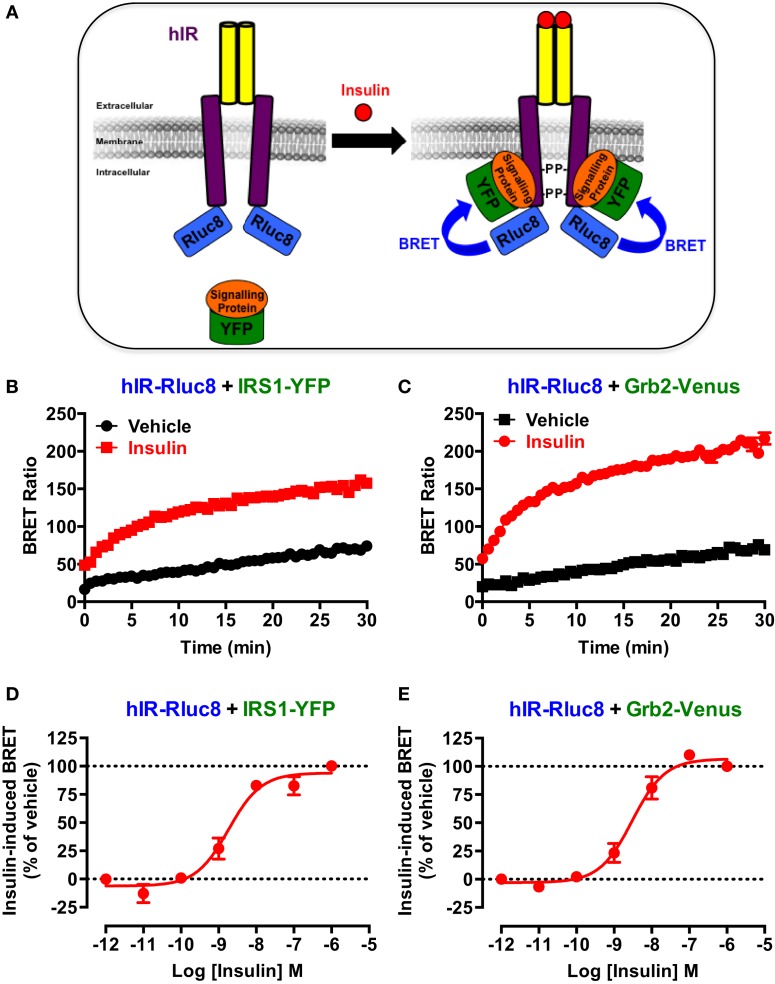
**BRET assay to monitor hIR activation**. **(A)** Schematic representation of the BRET-based assay to monitor insulin-induced hIR activation through the detection of the physical proximity between hIR–Rluc8 and its YFP-tagged signaling proteins. For this, HEK293 cells transiently co-expressing hIR–Rluc8 with either IRS1–YFP **(B,D)** or Grb2–Venus **(C,E)** were stimulated (red circle) or not (black square) with 100 nM **(B,C)** or increasing doses **(D,E)** of insulin and BRET measurements were performed in real-time and live cells as described in Section “Materials and Methods.” Data are mean ± SEM of three to six independent experiments performed in triplicate.

First, we wanted to validate the applicability of BRET approach to investigate the activation of hIR as previously reported for this receptor (23, 24) as well as EGFR (25) and various GPCRs (26–28). This is mostly based on agonist-promoted recruitment of cytosolic signaling proteins to the membrane receptor reflecting its activation. For this, Rluc8-tagged hIR and its interacting signaling proteins, IRS1 and Grb2, tagged with yellow fluorescent protein (IRS1–YFP) or Venus (Grb2–Venus), were used as BRET donor and acceptor, respectively (Figure 1A). The proteins were transiently and correctly expressed in HEK293 cells (data not shown) and BRET measurements were performed, in real-time and intact cells, under basal condition and upon activation with insulin (Figure 1A). Real-time analysis showed that stimulation of cells with 100 nM of insulin significantly promoted BRET increase between hIR–Rluc8 and IRS1–YFP (Figure 1B) and Grb2–Venus (Figure 1C). This nicely occurred in time-dependent manner with a sustained plateau even after 30 min, demonstrating the direct or indirect binding of the proteins to the activated hIR. The insulin-induced BRET increase occurred in a dose-dependent manner with the potency of insulin for hIR–IRS1 (*EC_*50*_* = *2.2 ± 0.6 nM, n* = *6*) (Figure 1D) and hIR–Grb2 (*EC_*50*_* = *4.4 ± 1.5 nM, n* = *6*) (Figure 1E) associations similar to what was previously reported (23, 29, 34), indicating insulin-promoted hIR activation. These data clearly demonstrate the applicability of BRET approach to assess insulin-induced hIR activation in real-time and live cells.

### Camel Milk Potentiates Insulin Action on Its Receptor

**Figure 2:**
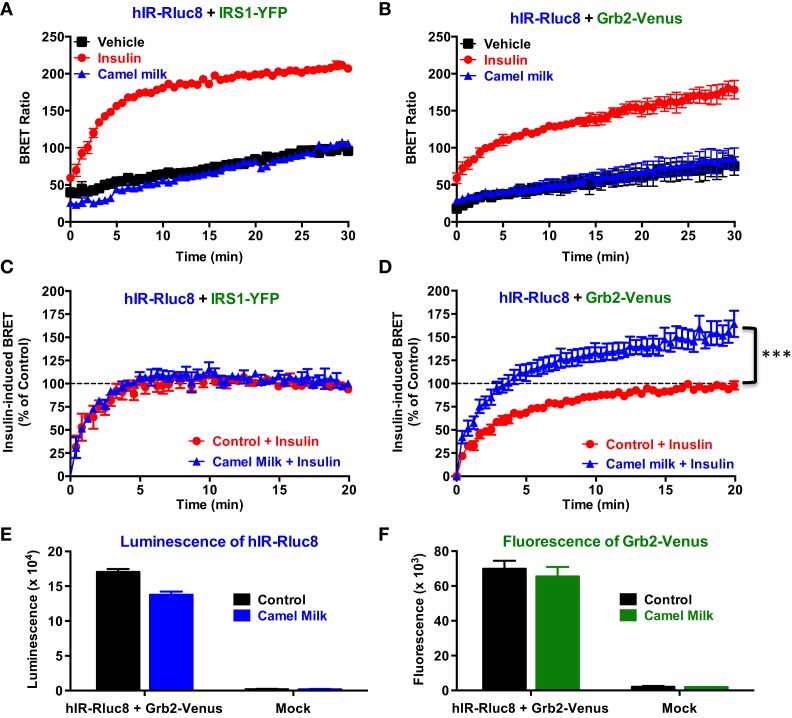
**Effect of camel milk on BRET between hIR–Rluc8 and its signaling proteins**. HEK293 cells transiently co-expressing hIR–Rluc8 and either IRS1–YFP **(A,C)** or Grb2–Venus **(B,D–F)** were first pre-treated (blue triangle) or not (black square and red circle) 30 min with camel milk before BRET measurements were performed in the absence or presence of stimulation with 100 nM of insulin as indicated. In parallel, luminescence **(E)** and fluorescence **(F)** analysis were carried out to quantify the relative expression of hIR–Rluc8 and Grb2–Venus, respectively. In **(C,D)**, insulin-induced BRET signals in cells not pre-treated with camel milk (red circle) were normalized to 100%. Data are mean ± SEM of 3–10 independent experiments performed in triplicate.

In order to investigate our hypothesis regarding the putative effects of camel milk on insulin and its receptor signaling, we examined the effect of camel milk on the association of IRS1 and Grb2 with hIR using BRET assay. Treatment of cells co-expressing hIR–Rluc8 and either IRS1–YFP (Figure 2A) or Grb2–Venus (Figure 2B) with camel milk did not promote any significant BRET increase compared to untreated cells (vehicle). By contrast, 100 nM of insulin nicely increased the BRET signals in both cases as expected (Figures 2A,B). This observation suggests no “insulin-like” effect of camel milk on hIR–IRS1 or hIR–Grb2 associations in HEK293 cells. Therefore, we examined the effect of camel milk on insulin action by treating cells 30 min with camel milk before their stimulation with 100 nM of insulin. Interestingly, while pre-treatment with camel milk did not change insulin-promoted BRET increase between hIR–Rluc8 and IRS1–YFP (Figure 2C), this significantly potentiated the effect of insulin to promote BRET increase between hIR–Rluc8 and Grb2–Venus (*146 ± 9%, n* = *10*, *at the plateau from 15 to 20 min of insulin stimulation*) (Figure 2D). Such an effect was not due to possible artifactual effects of camel milk on the luminescence of hIR–Rluc8 (Figure 2E) and fluorescence of Grb2–Venus (Figure 2F) that might result into non-specific BRET changes. Of course, the BRET increase induced by insulin likely reflects the recruitment of the cytosolic Grb2 to the activated hIR. However, camel milk may induce or stabilize specific conformation of hIR–Rluc8 leading to stronger proximity with Grb2–Venus and/or favorable orientation of the Rluc8 and Venus fluorophores following insulin stimulation without necessarily an increase in Grb2 binding. In all cases, camel milk-mediated potentiation suggests an allosteric interaction between camel milk components and insulin at the level of the receptor.

**Figure 3:**
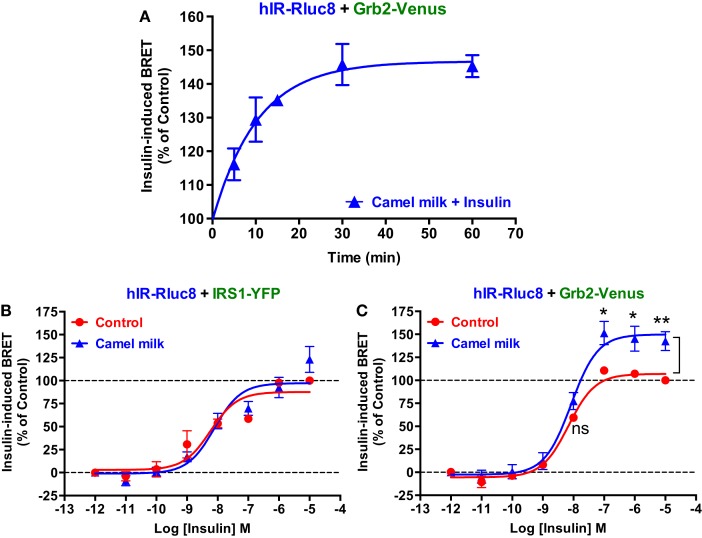
**Time-course and dose–response analysis of the effect of camel milk on BRET signals**. HEK293 cells transiently co-expressing hIR–Rluc8 and either Grb2–Venus **(A,C)** or IRS1–YFP **(B)** were first pre-treated (blue triangle) or not (red circle) different times **(A)** or 30 min **(B,C)** with camel milk before BRET measurements were performed in the absence or presence of stimulation with 100 nM **(A)** or increasing doses **(B,C)** of insulin as indicated. Data are mean ± SEM of three **(A,B)** or six **(C)** independent experiments performed in triplicate.

Next, we performed time-course as well as dose–response analysis on the effect of camel milk on BRET signals. Kinetic analysis on BRET between hIR–Rluc8 and Grb2–Venus showed a time-dependent effect of camel milk with a maximal effect from 30 min of pre-treatment (*145 ± 6%, n* = *3*, *at 30 min*) (Figure 3A). Then, dose–response analysis was performed in the absence and presence of treatment 30 min with camel milk that clearly showed that camel milk had no effect on the insulin dose–response in cells co-expressing hIR–Rluc8 and IRS1–YFP (Figure 3B). By contrast, camel milk significantly potentiated the maximal response (efficacy) of insulin in cells co-expressing hIR–Rluc8 and Grb2–Venus (*E_*max*_* = *142.6 ± 10.4%, n* = *6*, *at 10* μ*M of insulin*) (Figure 3C). Notice that no effect on insulin potency was observed, suggesting no effects on the binding properties of insulin receptor (Figure 3C). However, the fact that camel milk potentiated BRET signals at saturating concentrations of insulin further suggests an allosteric action, which seems to specifically impact hIR–Grb2 but not hIR–IRS1 association, conformation, and/or activation.

### The Peptide/Protein Nature of the Potentiating Agent of Camel Milk

**Figure 4:**
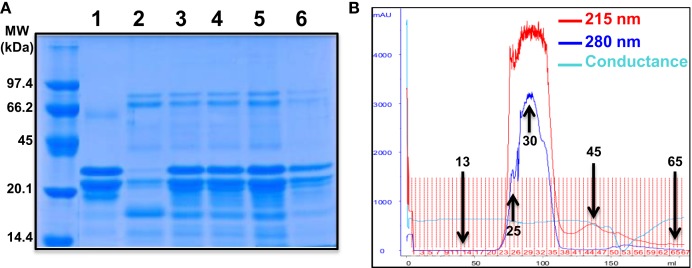
**Camel milk fractionations**. **(A)** The defatted camel milk was fractionated and the resulting fractions were submitted to SDS-PAGE followed by staining with the Coomassie Blue as indicated in Section “Materials and Methods.” The different tracks represent caseins (1) and whey (2) as well as the fractions 25 (3), 30 (4 and 5), and 45 (6) obtained from gel filtration chromatography **(B)**. For gel filtration chromatography, fresh camel milk was first defatted and passed through Sephadex G-25 column and different proteins and non-protein fractions were eluted according to their absorbance at 215 nm (red graph) and 280 nm (blue graph) and conductance for non-protein fractions (light blue).

**Figure 5:**
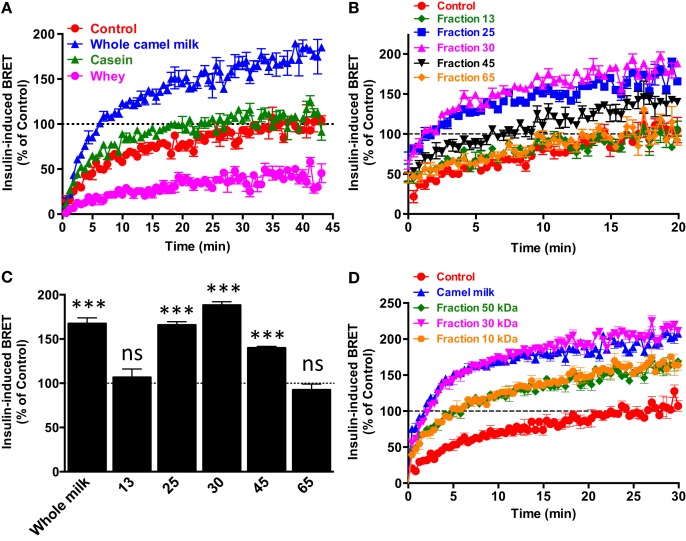
**Effect of camel milk fractions on BRET signals**. HEK293 cells transiently co-expressing hIR–Rluc8 and Grb2–Venus were first pre-treated or not (control, red circle) 30 min with either whole camel milk (blue triangle) or its different fractions obtained by either centrifugation **(A)**, gel filtration chromatography **(B,C)**, or molecular weight cutoff using appropriate filters **(D)**. Real-time BRET measurements were then performed in the absence or presence of stimulation with 100 nM of insulin as indicated. **(C)** The comparison of the maximal values of insulin-induced BRET averaged from the plateau of the curves shown in **(B)**. Data are representative of three independent experiments **(A,D)** or mean ± SEM of three experiments **(B,C)** all performed in triplicate.

To further characterize the potentiating effect of camel milk on insulin and its receptor, we performed various camel milk fractionations and tested their putative effects on BRET signals between hIR–Rluc8 and Grb2–Venus (Figure 4). First, we simply separated the whole milk into two major fractions, caseins that represent the major proteins (~80%) of the mammalian milk and whey containing the rest of milk proteins as illustrated by SDS-PAGE followed by Coomassie blue staining (Figure 4A). As shown in Figure 5A, while the whole defatted camel milk nicely potentiated insulin-induced BRET increase neither casein nor whey fractions (1 mg/ml) affected the insulin-induced BRET signals. The whey fraction had even a negative effect on the BRET signals (Figure 5A) probably due to the low pH (~5) of the fraction (data not shown). These data indicate that caseins cannot be involved and/or this method of fractionation did not allow preserving the biological activity of the camel milk.

Next, we separated the protein and non-protein fractions of camel milk using gel filtration chromatography through Sephadex G-25 column (Figure 4B). Protein quantification in the fractions that we randomly took revealed that significant proteins were collected in the fractions 25 (5 mg/ml), 30 (11.4 mg/ml), and to lower extent 45 (0.73 mg/ml). The presence of proteins in these fractions was also confirmed by SDS-PAGE followed by Coomassie blue staining (Figure 4A). These fractions freshly obtained were then tested for their effects on the BRET signals between hIR–Rluc8 and Grb2–Venus by taking the fractions 13 and 65 as non-protein ones. For this, cells were first pre-incubated 30 min with the different fractions and BRET signals were measured upon cell stimulation with 100 nM of insulin. As shown in Figures 5B,C, the potentiation of insulin-induced BRET signals was observed with the fractions 25, 30, and to lower extent 45, but not with the non-protein fractions, 13 and 65. These observations strongly suggest the involvement of peptides/proteins of camel milk in the potentiation of insulin-induced hIR activation.

To further consolidate this conclusion, we carried out separation of camel milk proteins according to their molecular weight using Amicon Ultra-15 centrifugal filters by cutting off at 10, 30, and 50 kDa and then tested the filtered fractions for their effects in BRET assay as described above. As shown in Figure 5D, similarly to the whole defatted camel milk, all the separated fractions significantly increased insulin-induced BRET signals. The difference in the effects may be due to the final proteins collected in each fraction after filtration (Figure 5D). Thus, these data further support the peptide/protein nature of the potentiating agent contained in camel milk. Moreover, the fact that the potentiation of insulin response was observed even with the preparation resulted from 10 kDa cutoff suggests that the potentiating agent has a molecular weight lower than 10 kDa. It is noteworthy to mention that all the effects were observed only with the freshly collected milk and separated fractions since such analysis using camel milk stored for more than a week did not show any effect of the whole milk or its fractions (data not shown). Together these data confirm the peptide/protein nature of the agent behind the biological activity of camel milk characterized by the potentiation of insulin-promoted hIR activation.

### The Effect of Camel Milk on hIR Downstream Signaling Pathways

**Figure 6:**
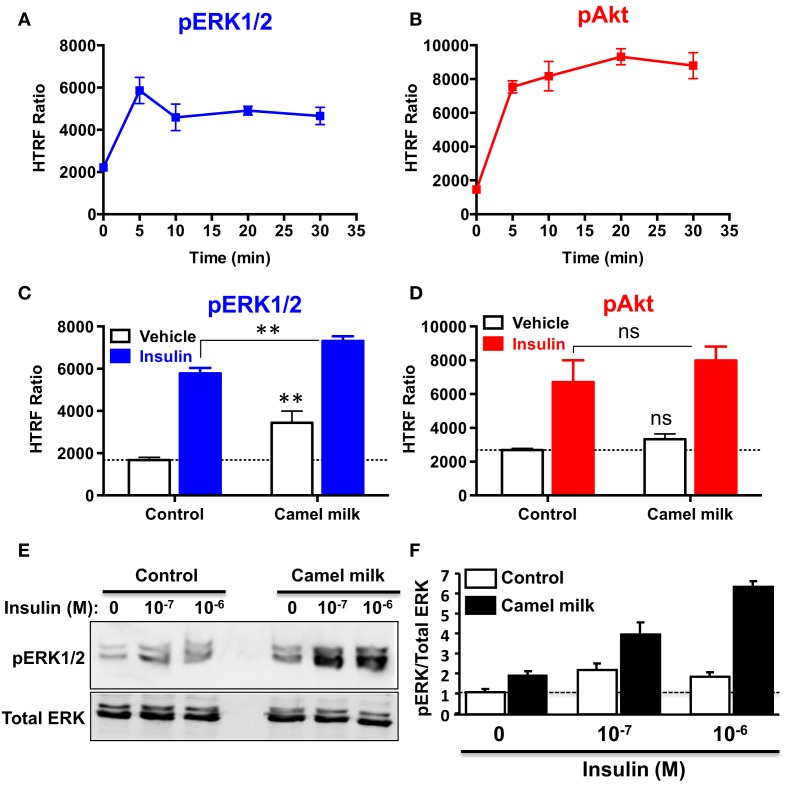
**Effect of camel milk on ERK1/2 and Akt phosphorylation**. HEK293 cells transiently co-expressing hIR–Rluc8 and Grb2–Venus were used for ERK1/2 and Akt phosphorylation using the HTRF^®^-based assay **(A–D)** and SDS-PAGE/western blot technique **(E,F)**. First, time-course analysis of phospho-ERK1/2 **(A)** and phospho-Akt **(B)** upon stimulation with 100 nM of insulin at different times was performed and HTRF signals were measured as described in Section “Materials and Methods.” Next, cells were first pre-treated or not 30 min with camel milk and stimulated or not 5 min with 100 nM of insulin before HTRF signals were measured as indicated. For the western blot analysis, cells were first pre-treated or not 30 min with camel milk and stimulated or not 5 min with 100 or 1 μM of insulin before SDS-PAGE and western blot using a rabbit polyclonal antibody against phospho-ERK1/2 (Thr202/Tyr204) **(E)**. The western blot signals were quantified and expressed in arbitrary units after normalization **(F)**. The HTRF data are mean ± SEM of three (for Akt) and four (for ERK1/2) independent experiments performed in triplicate. The western blot data are representative of two experiments.

To further investigate the effects of camel milk on hIR activation, we attempted to link our BRET data with the downstream signaling pathways mediated by insulin and its receptor. For this, we investigated ERK1/2 and Akt phosphorylation in HEK293 transiently expressing hIR–Rluc8 and Grb2–Venus using two approaches, an HTRF^®^-based assay in 384-well plate format as previously shown (32) and the classical SDS-PAGE followed by western blot. First, we performed a time-course analysis using HTRF^®^-based assay to determine the incubation time leading to the maximal ERK1/2 and Akt activation using the same cell samples. As a result, insulin nicely induced ERK1/2 phosphorylation with a transient kinetics and a maximum at 5 min of stimulation (Figure 6A). In addition, Akt phosphorylation occurred after 5 min of insulin stimulation but with a sustained response even after 30 min (Figure 6B). Therefore, we performed the following HTRF experiments at 5 min of stimulation with insulin that showed strong increase in the HTRF signals reflecting ERK1/2 (Figure 6C) and Akt (Figure 6D) phosphorylation mediated by hIR as previously shown for other receptors using similar HTRF^®^-based assay (32). Of course, these results are consistent with the BRET data showing strong insulin-induced BRET increase between hIR–Rluc8 and IRS1–YFP and Grb2–Venus (Figures 1 and 2) since it is well known that ERK1/2 and Akt activation through hIR specifically engages Grb2/Ras and IRS1/PI3-kinase pathways, respectively (29, 30).

Interestingly, we observed that treatment of cells 30 min with camel milk significantly increased the basal level of ERK1/2 phosphorylation and such effect was additive to the insulin-mediated response (Figure 6C). By contrast, neither the basal nor the insulin-promoted Akt phosphorylation was significantly affected by camel milk treatment (Figure 6D). To further investigate this aspect, we also examined ERK1/2 phosphorylation by SDS-PAGE followed by western blot using the specific anti-phospho-ERK1/2 antibody and stimulation with insulin at two saturating concentrations (100 nM and 1 μM). As shown in Figure 6E, camel milk treatment significantly increased the basal phospho-ERK1/2 and very largely potentiated insulin-dependent response with stronger potentiation at 1 μM of insulin as illustrated by the quantification of duplicated experiments (Figure 6F). By contrast, the phosphorylation of Akt assessed in parallel to ERK1/2 showed no significant effect of camel milk (data not shown). Together these observations are consistent with the HTRF data shown in Figures 6C,D using 100 nM of insulin. Interestingly, at 1 μM of insulin the cells pre-treated with camel milk showed a response being more than additive (sixfolds increase) compared to the sum of responses from untreated cells (twofolds) and milk-treated cells without insulin stimulation (twofolds) (Figure 6F). Even though, this observation does not clarify if camel milk induced ERK1/2 phosphorylation via hIR, this supports the existence of an allosteric effect of camel milk on insulin and its receptor with respect to ERK1/2 phosphorylation, which may depend on the allosteric potentiation of hIR–Grb2 association/activation observed in Figures 2 and 3.

## Discussion

As stated above, many previous studies *in vivo* using type 1-­diabetic rat models reported hypoglycemic properties of camel milk (11–19, 35). Because of the pivotal role of insulin receptor and its intracellular signaling pathways in the control of glucose uptake and its blood levels, we hypothesized that the hypoglycemic effects of camel milk may involve an action of its components directly on insulin receptor in insulin-dependent or -independent ways. To address this question, we examined the effect of camel milk and its fractions on the activation of hIR in HEK293 cells. For this, we used BRET technology based on the assessment of the physical binding of the two major key signaling proteins, IRS1 and Grb2, to hIR upon insulin stimulation as previously shown for hIR (23, 24) and EGFR (for Grb2) (25). Moreover, we attempted to link the BRET data with the downstream signaling of hIR through the study of the impact of camel milk on the two major hIR signaling pathways, including ERK1/2 and Akt phosphorylation in HEK293 cells.

Both real-time kinetics and dose–response BRET analysis clearly showed significant and specific recruitment of IRS1 and Grb2 proteins to the activated hIR with the expected potency of insulin (23, 29, 34). In addition, the BRET data showing strong insulin-induced BRET increase between hIR–Rluc8 and IRS1–YFP and Grb2–Venus (Figures 1 and 2) were also supported by the data demonstrating ERK1/2 and Akt phosphorylation via hIR activation in HEK293 cells (Figure 6). Together, these observations are in accordance with the classical paradigm of hIR signaling since it is well known that ERK1/2 activation engages Grb2/Ras pathway, while Akt activation results from the engagement of IRS1/PI3-kinase pathway (29, 30). Thus, our data validate the BRET assay by demonstrating that insulin-promoted BRET increases between hIR and IRS1 and Grb2 likely reflects the activation of hIR.

Next, we examined the effect of camel milk and its fractions on BRET signals between hIR–Rluc8 and either IRS1–YFP or Grb2–Venus. Indeed, in a direct treatment of cells with fresh camel milk alone, our data showed that camel milk did not promote any significant BRET increase between hIR–Rluc8 and IRS1–YFP or Grb2–Venus, suggesting that camel milk itself did not activate hIR in our model. It is worth noting that BRET is a proximity-based assay where the distance and the orientation of the donor (Rluc8) and the acceptor (YFP or Venus) are critical to make possible and efficient enough the energy transfer between Rluc8 and YFP/Venus within protein complexes. Accordingly, the absence of BRET increase between hIR–Rluc8 and IRS1–YFP and Grb2–Venus may have two different explanations. The first explanation implies the absence of hIR activation by camel milk and thereby no IRS1 and Grb2 recruitment is promoted. If such scenario happened, then our BRET data suggest the absence of any sufficient insulin in camel milk and/or “insulin-like” activity. The second possibility is that camel milk could to some extent activate hIR but the conformation of the activated hIR–Rluc8 was not translated into BRET changes with IRS1–YFP and Grb2–Venus. This means that the two proteins could bind to hIR but their relative amount is still weak and/or their distance and/or orientation are unfavorable within the activated complex to trigger sufficient BRET increase. Interestingly, in ERK1/2 phosphorylation assay, camel milk significantly induced ERK1/2 but not Akt phosphorylation even in the absence of insulin stimulation (Figures 6C,E), suggesting selective activation of ERK1/2 but not Akt pathway in HEK293 cells. Of course, such an effect could not be necessarily specific to hIR activation induced by camel milk since no evidence for camel milk binding directly to hIR. Thus, our data cannot rule out the possibility that camel milk activates other membrane receptors in HEK293 cells to induce ERK1/2 phosphorylation. Taken together with the BRET data, this point is still not clear and further investigations are required.

**Figure 7:**
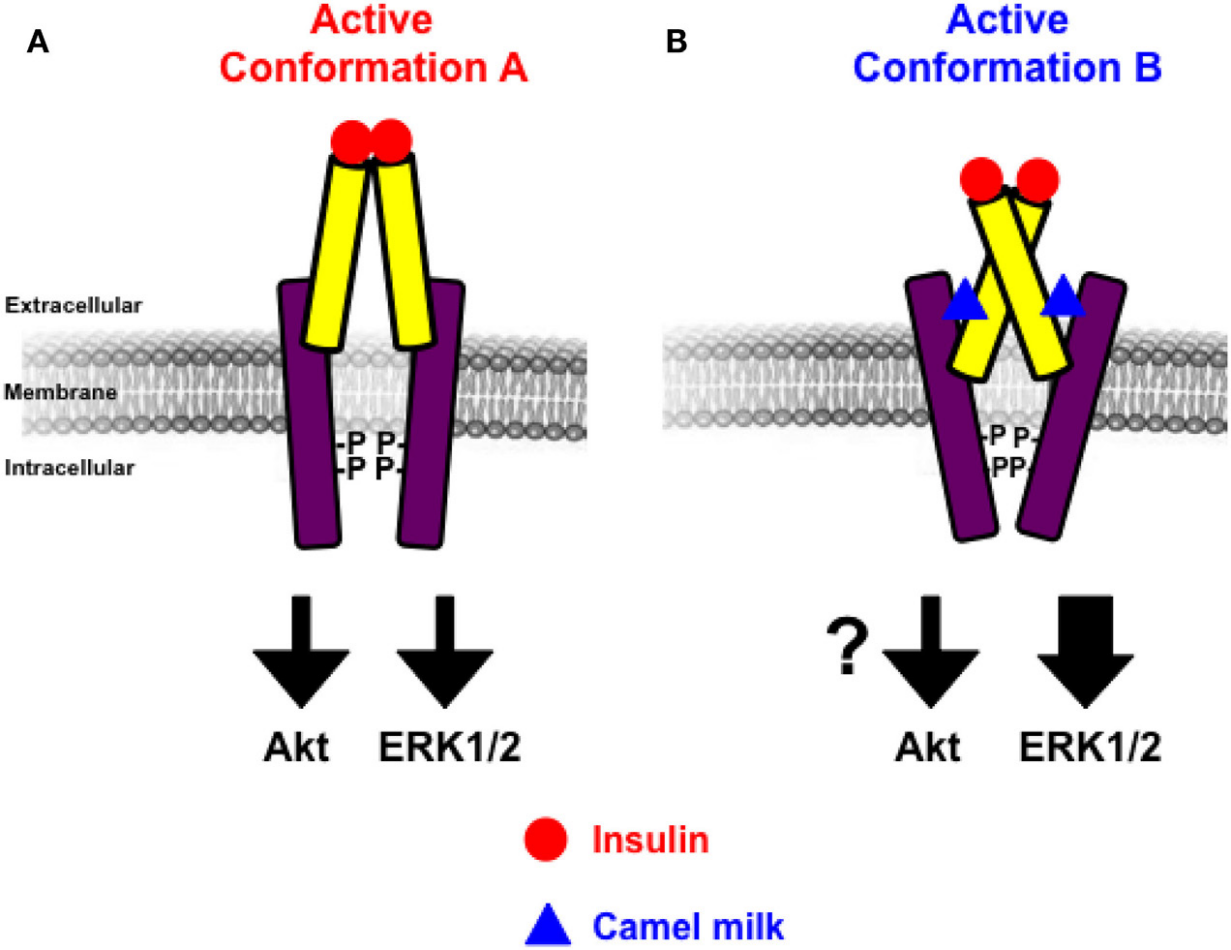
**Schematic model of allosteric action of camel milk on hIR**. The positive allosteric action of camel milk implies induction/stabilization of specific conformation of hIR with an impact on its downstream signaling. Indeed, in the presence of camel milk the conformation of hIR (*conformation B*) **(B)** is more efficient with regard to insulin-promoted ERK1/2 activation, but probably not Akt, compared to the conformation bound to insulin in the absence of camel milk component (*conformation A*) **(A)**.

The other important finding of our study is doubtlessly the effects of camel milk and the pharmacological profiling of its fractions on insulin-induced hIR activation. Our study constitutes the first demonstration of a direct effect of camel milk on hIR activation at the cellular level. Indeed, we observed that camel milk and some of its fractions intriguingly potentiated the efficacy but not the potency of insulin to promote BRET increase. Interestingly, this was specific to BRET between hIR–Rluc8 and Grb2–Venus, but not IRS1–YFP. Indeed, kinetics (Figure 2D) and dose–response (Figure 3C) analysis clearly demonstrated that camel milk selectively potentiated the BRET signal between hIR–Rluc8 and Grb2–Venus even at the maximal levels and the saturating concentrations of insulin (100 nM–10 μM). It is worth noting that camel milk did not affect insulin potency, suggesting no effect on the binding properties of insulin. This scenario is characteristic of an allosteric interaction between a camel milk component and insulin at the level of hIR, which specifically impacts hIR–Grb2 but not hIR–IRS1 association and/or conformation as well as the activation of the complex. Indeed, under our conditions, the activation level of hIR by insulin tends to saturation as shown in the dose curves. Subsequently, any further increase in the BRET signals cannot be explained by further recruitment of Grb2 to the activated receptor. The other argument for allosteric effects of camel milk is the absence of desensitization of hIR following 30 min of treatment with camel milk where insulin fully activated hIR and even a potentiation was observed. An orthosteric binding of camel milk would have been characterized by either no further activation by insulin due to the saturation of the system and/or desensitization of hIR or even an inhibition (in case of antagonistic action). One way to reconcile all this is to hypothesize that camel milk allosterically induces and stabilizes specific conformation of hIR, which may be different to both inactive (basal condition) and active (insulin bound) hIR (Figure 7). Such a conformation appears to be unfavorable with regard to BRET process between hIR–Rluc8 and IRS1–YFP and Grb2–Venus in the absence of stimulation with insulin. Therefore, orthosteric binding of insulin activates hIR by inducing further conformational changes within the receptor leading to favorable distance/orientation of both hIR–Rluc8 and Grb2–Venus (but not IRS1–YFP) and thereby causing BRET increase. Accordingly, if camel milk really induced ERK1/2 activation via hIR, this would support the existence of camel milk-specific conformation of hIR, which is able to engage Grb2 to the receptor and still sensitive to insulin stimulation even at the saturating concentrations through an allosteric activation mechanism. Of course, based on our data, the question whether camel milk induced ERK1/2 phosphorylation through its allosteric action on hIR is still unsolved. However, in camel milk-treated cells the insulin-mediated ERK1/2 phosphorylation was significantly potentiated in both HTRF^®^-based and western blot assays. Notice that the HTRF data only showed additive effects of camel milk on insulin-dependent ERK1/2 phosphorylation, indicating no direct association between hIR-dependent and -independent responses of the camel milk. By contrast, the western blot clearly demonstrated a strong potentiation of ERK1/2 phosphorylation that was more than additive in cells co-treated with camel milk and insulin (Figures 6E,F). This observation supports the existence of an allosteric effect of camel milk on ERK1/2 phosphorylation, which may depend on the allosteric potentiation of the conformation of hIR–Grb2 complexes as shown in Figures 2 and 3. Such allosteric modulation on insulin receptor has been recently reported when selective antibodies were used in combination with insulin stimulation arguing for the existence of functional allosteric binding sites on hIR (36, 37). Regarding the interaction of hIR with IRS1 and Akt phosphorylation, camel milk affected neither the BRET signal between hIR–Rluc and IRS1–YFP nor Akt activation, both the basal and insulin-mediated response. These observations suggest selective allosteric modulation of hIR by camel milk leading to changes in hIR–Grb2 interaction/conformation and ERK1/2 but not IRS1/Akt pathways. This is consistent with the concept of functional selectivity, also referred as biased signaling, which consists of a given hormone or agent activating one specific subset of receptor signaling without affecting others. Similar properties were reported on insulin-like growth factor-1 with respect to ERK1/2 relative to Akt pathways (38). Moreover, the concept of biased signaling is now well documented for other membrane receptors, such as GPCRs (39, 40).

Furthermore, the data on BRET between hIR–Rluc8 and Grb2–Venus using the whole camel milk were also supported by those with the fractionated milk where the defatted milk was submitted to different separation protocols. Together, the fractionation data converged to suggest that the potentiating agent contained in camel milk has a peptide/protein nature with a positive effect also observed in the fraction <10 kDa. Our data may be consistent with the previous studies showing that the amino acid sequences of some of the camel’s milk proteins are rich in half-cystine similarly to peptides belonging to insulin family (20). Of course, this crucial aspect needs further experimental and technical analysis and improvement in the aim to obtain optimal fractionation and identify the real potentiating agent. Also, we observed that the stability and storage condition of camel milk are two important elements determining the functionality of camel milk as previously shown for camel milk insulin (4). The activity of camel milk or its resulted fractions was significantly decreased or totally abolished when camel milk was used after its long storage further demonstrating the biological activity of camel milk components (data not shown). Together, our data are of great importance in the context of chemical and biochemical profiles of camel milk compared to other related milks, such as bovine or goat (5, 41–43). Indeed, the question regarding insulin content of camel milk is still unclear since some studies reported that camel milk contains high levels of insulin (2, 44), while others showed low insulin levels but significantly higher in camel colostrum (45). Moreover, insulin concentrations in camel milk have been reported to significantly depend on the lactation stages (3) as well as temperature and storage conditions (4, 46). These different observations must be considered when interpreting the studies reporting the hypoglycemic effects of camel milk *in vivo* using both animal and human models of type 1 diabetes (11–19, 35). In fact, it still not clear how to explain the beneficial effects of camel milk and its plausible content of insulin since the hormone should be degraded in the stomach and if not it has to remain functionally intact during the absorption process in the intestine (22, 45).

From the pathophysiological point of view, our findings in transfected HEK293 cells using BRET technology help to better understand the previous studies on the hypoglycemic properties of camel milk in both animal and human models (11–19, 35). Indeed, our data bring for the first time the evidence for camel milk acting allosterically at the level of insulin receptor that appears to differentially impact its downstream signaling (Figure 7). Nevertheless, the link between the effects of camel milk that we reported here and its hypoglycemic properties is still weak since our data reported potential effects on the mitogen-activated protein kinase (MAPkinase) involved in proliferation and differentiation processes of hIR (29, 30). By contrast, no significant effects were observed on hIR–IRS1 association and Akt phosphorylation constituting the metabolic pathway involved the transport of glucose and glycolysis (29, 30). Therefore, further investigations are required to confirm our data in other models and to better dissect such effects of camel milk components at the molecular level in order to make a link with putative effects on glucose uptake in insulin-sensitive tissues. Also, the big challenge would be the identification and characterization of the active agent contained in camel milk and investigating its effects on insulin binding and hIR activation. The implication of adiponectins and other hormones contained in milk and reported to influence hIR activity and signaling could be the interesting tracks to investigate (47–49). Finally, it would be interesting to extend the study to other receptors involved in the glucose homeostasis.

## Author Contributions

AA performed most of the experiments and analyzed the data. MI performed the fractionation of camel milk. KA-H helped in BRET and HTRF experiments. AA-S designed and supervised some aspects of the project. CR and JD performed the western blot and expertise in insulin signaling. MA conceived and supervised the project, performed some BRET experiments, analyzed the data, and wrote the manuscript.

## Conflict of Interest Statement

The authors declare that the research was conducted in the absence of any commercial or financial relationships that could be construed as a potential conflict of interest.
